# β-hydroxybutyrate and ischemic stroke: roles and mechanisms

**DOI:** 10.1186/s13041-024-01119-0

**Published:** 2024-07-29

**Authors:** Ge Feng, Zongkai Wu, Leyi Yang, Kaimeng Wang, Hebo Wang

**Affiliations:** 1 Graduate School of Hebei Medical University, Shijiazhuang, Hebei China; 2 Department of Neurology, Hebei General Hospital, No. 348 21 Heping West Road, Shijiazhuang, 050051 Hebei China; 3 Hebei Provincial Key Laboratory of Cerebral Networks and Cognitive Disorders, Shijiazhuang, Hebei China

**Keywords:** β-hydroxybutyrate, Ischemic stroke, Prognostic biomarker, Neuroprotective effect

## Abstract

Stroke is a significant global burden, causing extensive morbidity and mortality. In metabolic states where glucose is limited, ketone bodies, predominantly β-hydroxybutyrate (BHB), act as alternative fuel sources. Elevated levels of BHB have been found in the ischemic hemispheres of animal models of stroke, supporting its role in the pathophysiology of cerebral ischemia. Clinically, higher serum and urinary BHB concentrations have been associated with adverse outcomes in ischemic stroke, highlighting its potential utility as a prognostic biomarker. In both animal and cellular models, exogenous BHB administration has exhibited neuroprotective effects, reduction of infarct size, and improvement of neurological outcomes. In this review, we focus on the role of BHB before and after ischemic stroke, with an emphasis on the therapeutic potential and mechanisms of ketone administration after ischemic stroke.

## Background

Stroke is the second leading cause of global morbidity and mortality, second to ischemic heart disease [1]. Furthermore, It is also the foremost cause of long-term disabilities [2]. Currently, stroke research is mainly focused on ischemic stroke, which accounts for 87% of all stroke cases [3]. Atherosclerosis is the chief instigator of ischemic stroke incidence among the underlying causes. Reducing stroke incidence and improving patient prognosis is an urgent and compelling challenge in the scientific community.

In conditions of carbohydrate scarcity, fats are converted into fatty acids, which are then metabolized in the liver to produce ketone bodies [4]. Ketone bodies include β-hydroxybutyrate (BHB), Acetone, and Acetoacetate. As research on ketone bodies continues to advance, their application in the treatment of neurological disorders has become a major focus [5, 6].

BHB, making up about 70% of the circulating ketone body pool [7], has garnered interest in the fields of metabolism and biological regulation as a major physiological ketone substance [8]. BHB has been identified as a high‑frequency metabolic biomarker of IS and elevated level of BHB has been associated with poor prognosis in IS patients. However, the neuroprotective mechanisms mediated by ketone bodies within the context of cranial neural networks remain incompletely understood. Current research focuses on excitotoxicity, oxidative stress, autophagy, blood-brain barrier, and epigenetics. We propose an updated theoretical framework to guide future stroke prevention research and improve patient outcomes. Additionally, this paper will discuss the potential therapeutic implications of exogenous BHB supplementation in stroke management.

## The physiological effects of β-hydroxybutyrate

Ketone bodies, primarily derived from lipid metabolism, are produced through fatty acid oxidation. BHB has the highest concentration of ketone bodies in the blood. In essence, BHB acts as a unique substitute for glucose in cases of fuel supplies deficit in the brain [9]. The ketogenesis process has been systematically reported since 1958 and the production of ketone bodies is a dynamic process with several inputs and regulatory checkpoints [7, 10–12]. Ketogenesis mainly occurs in the mitochondria of the liver [8].

**Fig. 1:**
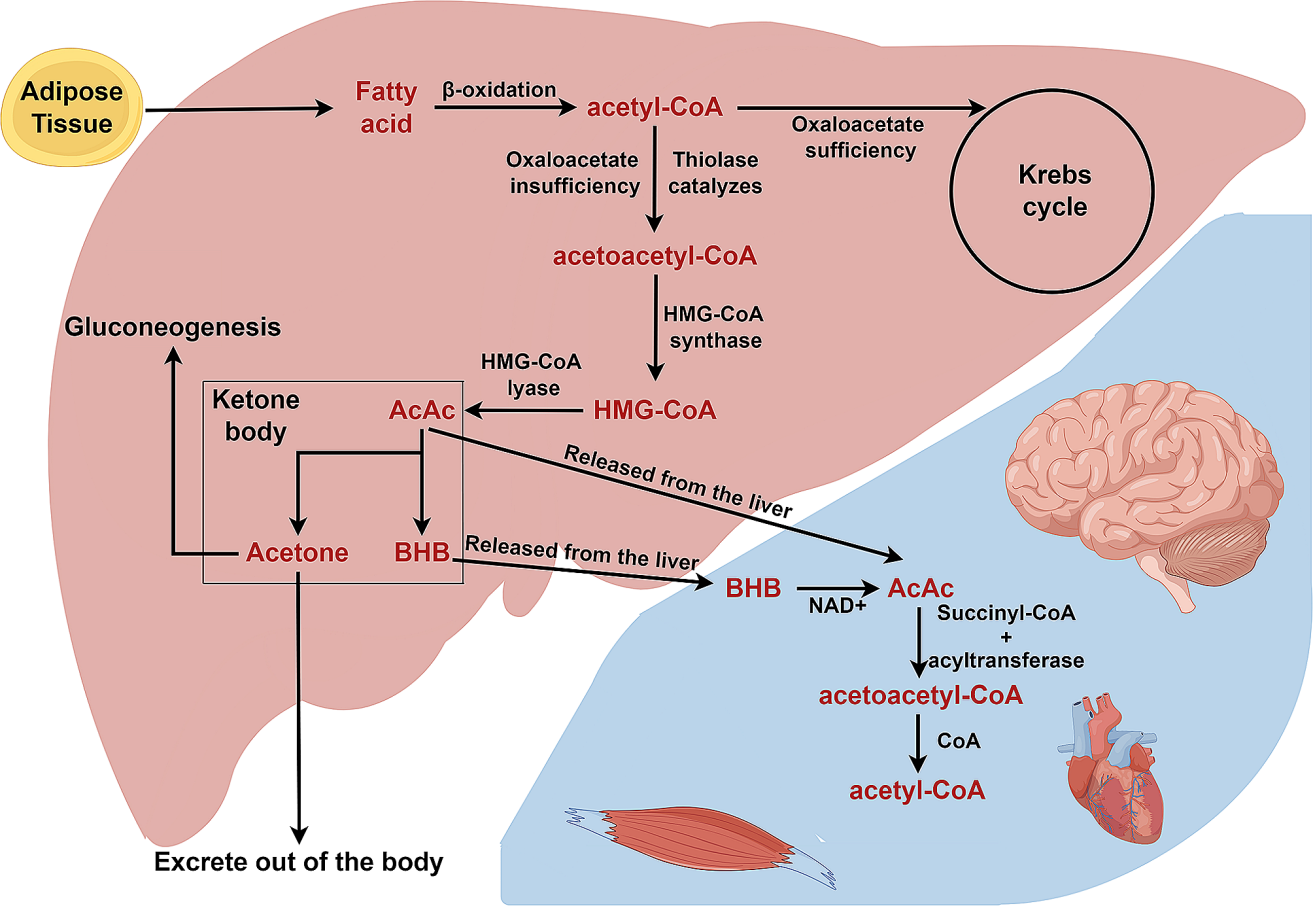
BHB synthesis and decomposition. Fatty acids are β-oxidized to acetyl-coA in the liver. When oxaloacetate is insufficient, acetyl-coA is converted to acetoacetyl-coA and then to HMG-CoA. HMG-COA can be converted to AcAc and subsequently to acetone or BHB. AcAc and BHB can be converted into acetyl-coA in a variety of human tissues after being released from the liver. This figure was drawn by Figdraw

Free fatty acids, mobilized from adipose tissue, are transported into hepatic mitochondria where they undergo β-oxidation to produce two molecules of acetyl-CoA. This process may be augmented by a ketogenic diet [13]. When mitochondria provide an insufficient amount of oxaloacetate to condense with acetyl-CoA to form citric acid and enter the Krebs cycle, thiolase catalyzes the condensation of acetyl-CoA to acetoacetyl-CoA. Subsequently, HMG-CoA synthase catalyzes the binding of a third acetyl-CoA molecule to produce HMG-CoA, a process intricately regulated and recognized as the key mechanism imparting temporal and spatial precision to BHB synthesis [14]. HMG-CoA is then cleaved to produce the unstable ketone body, AcAc, which is converted into stable BHB via β-hydroxybutyrate dehydrogenase. The monocarboxylate transporter proteins are thought to mediate the transport of BHB from the liver to peripheral tissues across the cell plasma membrane [15, 16]. As a small polar molecule, BHB is readily soluble in blood. BHB is metabolized back into acetyl-CoA within tissues, Acetyl-CoA enters the Krebs cycle for facilitating high levels of ATP production through oxidative phosphorylation. A portion of BHB also crosses the blood-brain barrier (Fig. 1).

Moreover, BHB stands out as a molecule with diverse functions that extend beyond its conventional role as an energy substrate [17]. One of the most intriguing aspects of BHB is its role as an endogenous and selective inhibitor of Class I histone deacetylases (HDACs). The regulation of histone acetylation modification affects downstream transcription, thereby inhibiting vascular calcification and other effects [18]. BHB engages with a spectrum of cellular receptors, including free fatty acid receptor 3 (FFAR3), hydroxycarboxylic acid receptor 2 (HCAR2), also known as G protein-coupled receptor 109 A (GPR109A) [8]. Notably, BHB’s inhibition of FFAR3 has been reported to exert anti-lipolytic effects by suppressing activity in the sympathetic nervous system [19], which may represent a cardiovascular benefit [20]. FFAR3 is known to couple with PTX-sensitive G-proteins, leading to voltage-dependent blockage of N-type Ca^2+^ channels in sympathetic neurons [21]. Conversely, BHB may directly suppress sympathetic nervous system activity through the antagonism of FFAR3 expressed in sympathetic superior cervical ganglion neurons [22]. The mechanism of another receptor will be detailed in the following text.

BHB has two enantiomers, D-β-hydroxybutyric acid(D-BHB) and L-β-hydroxybutyric acid(L-BHB). The proportion of D-BHB produced by the above ketogenic process accounts for 97-98%. There is a hypothesis that L-BHB is mainly converted into D-BHB in vivo [23]. And it is mainly D-BHB rather than L-BHB that participates in the tricarboxylic acid cycle and increases ATP levels [24]. Therefore, in the following text, the term BHB typically refers to D-BHB, with specific mention of the stereoisomer type in studies addressing the aforementioned chiral differences.

## BHB level in ischemic stroke patients

It is widely accepted that under hypoxic conditions, the brain shifts its metabolic preference towards the utilization of ketone bodies as a compensatory mechanism. Among these ketone bodies, BHB is particularly noteworthy, often referred to as a “biofuel” [25]. This ketone body enhances the respiratory capacity of neuronal mitochondria, thereby counteracting the inhibitory effects on glucose-based aerobic energy metabolism induced by cerebral ischemia. In the acute phase of ischemic stroke, the levels of BHB in brain tissue, blood, and urine are elevated. However, elevated levels of BHB in blood and urine are associated with poor prognosis (Table 1). Uncovering metabolic biomarkers associated with ischemic stroke susceptibility holds promise for advancing individualized prophylactic interventions and optimizing post-stroke patient prognosis in clinical settings.

**Table 1:** Levels of BHB in brain tissue, plasma, and urine of stroke patients and animal models of stroke.

Type of samples	Species	Lesions	Test method	Result	Reference
Brain tissue	mice	t-MCAO for 90 minutes VS blank control	Gas chromatography–mass spectrometry	increased	[27]
Blood	mice	t-MCAO for 90 minutes VS blank control	Gas chromatography–mass spectrometry	increased	[27]
Brain tissue	rats	MCAO for 2 hours VS blank control	^1^H NMR Spectroscopy	increased	[28]
Blood	Human	AIS VS controls	Nuclear magnetic resonance	increased	[30]
Blood	Human	24 hours after treatment with rt-PA	Nuclear magnetic resonance	High level BHB is associated with a 3-month poor functional outcome	[31]
Capillary blood	Human	Patients with first-ever acute stroke	Beta-Ketone Monitoring System	Higher value BHB was independently associated with lower odds of mRS 0–2	[33]
Urine	Human	AIS patients with positive urine ketone bodies VS patients with negative urine ketone bodies	Urinary dipstick test	Higher risk of poor outcome upon discharge	[36]
Urine	Human	AIS or TIA patients with positive urine ketone bodies VS patients with negative urine ketone bodie	Urine dipstick test	Elevated risk of all-cause mortality at 3 months and 1 year	[37]

t-MCAO: transient middle cerebral artery occlusion; AIS: acute ischemic stroke; TIA: transient ischemic attack; MCAO:middle cerebral artery occlusion; rt-PA: recombinant tissue plasminogen activator

### BHB in brain tissue: insights from animal models

During the acute phase of acute cerebral infarction, oxidative stress inhibits the Krebs cycle, activates anaerobic glycolysis and gluconeogenesis, and significantly increases the brain’s uptake of ketones to meet energy needs [26]. Due to the difficulty in obtaining human brain specimens, the brain tissue used in stroke research is generally derived from animal specimens. In metabolomics studies using rat cerebrospinal fluid, blood, and brain tissue as biological samples, the BHB in the stroke group showed a consistent increasing trend in the subgroups of brain tissue, while the other two did not. This observed trend is not influenced by the different modeling methods employed [27]. This suggests that attention should be paid to the potential role of BHB in the mechanism of acute ischemic stroke.

During the acute phase after ischemic stroke, mice on a normal diet showed an increase in BHB levels in the brain, a decrease in glucose levels, and a corresponding increase in BHB levels in the liver and blood. However, compared to mice fed a diet rich in fat, the improvement level was not significant [28]. After the improvement of energy metabolism through drug treatment, the BHB level in brain, as analyzed in metabolomics studies of middle cerebral artery occlusion (MCAO) rats, decreased significantly [29], indicating an improvement in brain energy metabolism.

### BHB in blood and its clinical implications

The concentration of BHB in the blood has been observed to vary at different stages of ischemic stroke, with an increased level during the acute stage, which may have implications for patient prognosis. A research team performed serum nuclear magnetic resonance (NMR) analysis on patients with acute ischemic stroke during both the acute phase, specifically within 72 h of stroke onset, and the chronic phase, spanning 3 to 6 months post-stroke. Their unpublished conclusions also mentioned that ketone bodies were higher in the acute stage of ischemic stroke than in the control group, while they decreased to the control group level in the chronic stage. Therefore, ketone bodies in stroke may be metabolites driven by acute cerebral ischemia rather than baseline changes in stroke patients [30], which is consistent with the conclusion drawn from animal experiments.

Although BHB fills the energy gap that occurs after a stroke, a high BHB level does not necessarily imply a good prognosis. A study examined metabolomic features of stroke patients and found that serum BHB in the acute phase of stroke was statistically significantly related to the development of poor functional outcomes (modified Rankin Scale, mRS = 3–6) at 3 month [31]. Patients who died within three months of a stroke had significantly higher levels of BHB during the acute phase compared to those who survived [32]. A prospective observational study in stroke patients indicated that an elevated capillary BHB concentration upon hospital admission was associated with an increased likelihood of a poor outcome three months post-stroke(mRS = 3–6) [33]. In studies investigating the metabolic characteristics of different IS subtypes, BHB did not demonstrate traits of significant clinical relevance [34, 35].

### BHB in urine: potential prognostic biomarker

Urinary ketone bodies may have more potential as biomarkers of IS. In another easily accessible body fluid: urine, the presence of BHB has been explored as a potential prognostic biomarker, with studies suggesting a correlation between urinary BHB levels and stroke severity. Participants without diabetes mellitus with positive urinary ketone bodies had a possibly more severe stroke (higher NIHSS score upon discharge) [36]. The mechanism is still unclear. Patients with positive urine ketone bodies have higher blood pressure and blood sugar, and this baseline difference may be related to their poor function. Another study, which includes more patients, suggests that urinary ketone positivity is associated with a poor long-term prognosis in stroke patients at 3 months and 1 year [37].

### Challenges and future directions

High BHB may be a negative predictor of stroke outcome, and urinary ketone bodies may have more potential as biomarkers. The underlying mechanism could be attributed to systemic inflammatory responses and heightened levels of oxidative stress induced by ketoacidosis [38].

In most of the studies mentioned above, patient groups were not matched for vital characteristics, and stringent exclusion criteria were not applied. Stroke risk factors such as diabetes, hypertension, dyslipidemia, and arterial disease confer distinct metabolic profiles. Therefore, considering the above factors, using metabolomics to study the role of BHB in cerebral infarction in stable and reproducible animal models may have greater guiding significance for clinical research.

Concurrently, due to the challenges in sample acquisition, it is unclear whether BHB concentrations in the cerebrospinal fluid and brain tissue can predict patient prognosis.

Additionally, while the majority of current research suggests that high BHB levels indicate a poor prognosis, it cannot be excluded that the nonlinear relationship between the two has not been fully explored. For instance, it is possible that we have only discovered a segment of an L-shaped or U-shaped curve. This hypothesis necessitates further investigation through clinical studies with larger sample sizes and more comprehensive indicators to explore the aforementioned assumptions [33]. After IS, the ketogenic response is activated, and high levels of BHB circulation may indicate high levels of cerebral ischemia and poor prognosis. The much higher levels of BHB caused by exogenous supplementation may play a neuroprotective role and help improve prognosis. The mechanism behind this hypothesis also needs further exploration.

## Effect of exogenous BHB supplementation

Intermittent fasting, adherence to low-carbohydrate ketogenic diets, or application of ingested exogenous ketones are all under investigation, with the objective of enhancing wellness and performance, improving health, combatting disease, and offsetting the effects of aging [39–42]. Choosing an appropriate time to initiate exercise is crucial after a stroke [43], as commencing physical activity too soon may potentially accelerate cerebral cell apoptosis [44, 45]. Implementing fasting programs targeting stroke patients is inhumane. Therefore, we focus on introducing two methods: ketogenic diet and exogenous supplementation of BHB preparation (Table 2).

**Table 2:** The effect of exogenous supplementation of BHB on stroke animal models.

Species	Supplement	Dosage of formulation	Administration time	Changes in neurological function	Reference
Rats	Classic KD plus MCT oil diets	Mixing fat to protein and carbohydrate with a 4 to 1 ratio and 3.5 cc/day MCT oil	3 days before stroke induction and 7 days after it	A significant reduction in motor-behavior impairment	[49]
Rats	Na-BHB injected into the lateral ventricle	10 mmol/L at a delivery rate of 1 ml/h	4 days before MCAO	Significant decreased in infarct size	[50]
Mice	KD diet	Macronutrient composition of mouse diets: 89.5% fat;10.4% protein ;0.1% carb	3 weeks before MCAO	KD significantly reduced infarct area	[51]
Mice	KD diet	Macronutrient composition of mouse diets:89.5% fat ;10.4% protein ;0.1% carb	4 weeks before MCAO	A proportional decrease in infarct volumes with increased blood BHB levels	[52]
Mice	KD diet	Fed a high fat, no carbohydrate diet	3 weeks before MCAO	KD significantly reduced infarct volume	[53]
Rats	BHB injected into the lateral ventricle	BHB low-does group: 250 µg/kg	Administer medication 1 hour after MCAO, and restore perfusion 2 hours later, in total 4 µL	Lower modified neurological severity scores and smaller infarct volumes were showed in all three groups	[100]
		BHB medium-does group: 500 µg/kg			
		BHB high-does group: 1000 µg/ kg			
Rats	DL-BHB injected intraperitoneally	500 mg/kg body weight	1 hour after stroke induction	Partial amelioration of the motor deficit	[102]
Mice	Sodium BHB injected intraperitoneally	30 mg/kg	Immediate administration upon reperfusion after MCAO for 90 minutes	The score of behavioral experiments improved significantly	[103]
Mice	BHB injected subcutaneously	0.4 mmol/kg	30 minutes after MCAO	Ketone treatment reduced infarct volume and improved neurologic function	[106]

KD: ketogenic diet; MCT: medium chain triglycerides; MCAO :middle cerebral artery occlusion

### Ketogenic diet

Intake of a large amount of fat increases fatty acid oxidation and accumulates ketone bodies [46]. A well-established method to elevate BHB levels in the body is through adherence to KD [42]. A ketogenic diet (KD) is characterized by high fat content, low carbohydrates, and adequate protein, originally aimed to treat epilepsy [47]. Recent years have witnessed the diet’s therapeutic potential for neurological diseases such as Parkinson’s disease and stroke [48]. Accumulating evidence suggests that KD can reduce infarct volume and improve neurological function after MCAO.

For inducing ketosis in experimental studies, commonly employed methods encompass acute interventions such as a combination of fasting for 12 h and a classical KD for three days [49], as well as subacute three-week KD [50, 51]. These methods exhibit protective effects on ischemic brain tissue, manifested as improving motor ability [49], reducing infarct volume [51, 52], increasing cerebral blood flow [53], and mitigating mitochondrial damage in stroke-induced mice [54]. Rats that had been fed the KD for 25 days before the injury exhibited resistance to neurodegeneration induced by cerebral ischemia following cardiac arrest [55], suggesting the potential improvement benefit of KD on ischemic stroke prognosis. Additionally, structural and functional plasticity is observed in the ipsilateral cortex, contralateral cortex, and corticospinal tract [56]. Due to our current inability to predict accurately the onset time of stroke, rendering the aforementioned ketosis induction methods inappropriate for translation into clinical research. However, research on the KD’s post-stroke protective effects is relatively scarce compared to pre-stroke studies [57]. The ketogenic state induced by caloric restriction after induced ischemia did not seem to play a role in brain protection and nerve recovery [58].

Therefore, adopting a ketogenic diet in high-risk populations of ischemic stroke may play a role in improving the prognosis after onset. Furthermore, KD may bolster metabolic health, enhancing insulin sensitivity, and attenuating inflammation [59], all of which are associated with the risk of stroke. Studies have demonstrated the safety of long-term KD in both healthy and high cholesterol individuals, with recorded significant weight loss and improved blood lipid levels [60]. Notably, participants in these studies gradually increased dietary carbohydrates to an optimal level and incorporated a 45-minute walk daily [61, 62]. MRI experiments in rats have shown that long-term use of a ketogenic diet alters the pattern of striatal connections and significantly modifies astrocyte-related metabolites [63], warranting further exploration into the diet’s long-term impact on brain physiological processes.

In conclusion, while a ketogenic diet presents promising avenues for stroke prevention, it necessitates professional guidance and should be complementarily paired with regular exercise. This balanced approach is likely to unlock the full preventive and therapeutic potential of the KD in ischemic stroke, necessitating comprehensive exploration and rigorous clinical trials.

### Exogenous supplementation of BHB preparations

Contrary to increasing fatty acid oxidation in a ketogenic diet, consuming ketone supplements can quickly increase circulating ketone body concentration and reduce fatty acid mobilization through negative feedback inhibition [46]. In addition to differences in mechanism, from the perspective of clinical application, exogenous supplementation of BHB with ketone preparations can accurately regulate BHB concentration within the treatment window, and the diet of the subjects is not restricted. Commonly used ketone preparations include ketone bodies (mainly BHB) or BHB precursors, such as oral or injection forms of ketone mineral salts, ketone esters and medium chain triglycerides (MCT).

The following section will discuss experiences from clinical trials involving the supplementation of exogenous ketone bodies. It is anticipated that clinical trials will soon emerge to bridge the existing gap between preclinical evidence and the neuroprotective effects in human stroke.

#### Sodium ß-hydroxybutyrate

Exogenous methods of inducing ketosis include oral or intravenous BHB delivery. Administration of a 300 mg/100 g body weight concentration of BHB sodium in rats increased ketonemia [64]. Administration of 7.5 g of BHB daily to healthy adolescents for a duration of up to three months has been demonstrated to be safe, without impacting key health indicators such as bone density [65]. A daily intake of 2.9 g of BHB over a 12-week period in healthy adult participants appears to have an effect on reducing body fat percentage [66]. But there have been no studies on administering BHB salts to stroke patients yet.

Intravenous injection of BHB has been shown to increase cerebral blood flow and reduce cerebral glucose consumption [67, 68]. In addition, due to the sudden onset of stroke, intravenous injection of BHB is more suitable for the above clinical scenarios. Due to reasons such as first-pass elimination, 76% of the oral volume was required to match BHB concentrations via the IV delivery methods [69]. The mineral salts of BHB commonly employed in intravenous BHB formulations consist of a racemic blend of D-BHB and L-BHB isoforms(D/L-BHB) and there is a linear relationship between blood BHB concentration and infusion rate [69]. Compared to the administration of D-BHB alone, despite a slightly lower ketone concentration, the duration of sustained ketosis significantly extend when consuming a racemic mixture [70]. L-BHB may accumulate in the body during consumption, but its signaling role in the body is not yet clear [71]. Therefore, intravenous injection of BHB is easier to control blood BHB concentration to reach the ideal range.

#### Ketone ester

Ketone esters (KE) are a general term for a class of compounds, being composed of one or more ketone bodies and alcohol components [72]. The current research focus is on cardiovascular diseases [73–75], and the field of cerebrovascular diseases still needs to be explored. Over the last decade, the oral ingestion of KE has been developed and they are now commercially available. KE is hydrolyzed into BHB and its precursor 1,3-butanediol, which may also be a potential analogue of BHB [76]. A simple ketone drink can rapidly increase blood BHB concentration, bypassing any dietary restrictions and avoiding increased acid or salt loads when exogenous Na-BHB or BHB acid forms are used [77]. Studies have shown that oral administration of ketone ester beverages for up to 28 days does not affect the physical or biochemical blood or urine parameters of healthy subjects [78], demonstrating that oral KE is a safe method to increase blood ketone body concentration. KE can control liver cholesterol biosynthesis by limiting the availability of sterol synthetic precursors, such as acetoacetyl-CoA and HMG-CoA [79], offering potential benefits to patients. When facing a sudden energy crisis, supplementing with KEs has also been shown to have anti-inflammatory effects [73]. In recent years, new ketone ester formulations, such as bis-hexanoyl (R)-1,3-butanediol [80], (R)-3-hydroxybutyl (R)-3-hydroxybutyrate, have been developed. A randomized controlled trial has demonstrated that the administration of KE solution in healthy adults is safe [81] and may have the potential to alter levels of Alzheimer’s disease pathological biomarkers [82]. In adults with obesity, 14 days of premeal KME supplementation improves glucose control, enhances vascular function, and may reduce cellular inflammation. KME supplementation may be a viable, nonpharmacological approach to improving and protecting vascular health in people with heightened cardiometabolic risk [83]. Moreover, after oral administration of KEs in mice, the levels of acetyl-CoA in the brain rapidly increase, promoting the citric acid cycle while inhibiting glycolysis [84]. Given these advancements, we have reason to anticipate the development and application of more and safer ketone ester preparations for the treatment of acute stroke patients.

#### Medium chain triglycerides (MCTs)

MCTs can be extracted from natural sources through lipid fractionation, such as coconut oil, palm oil, and butter. MCTs are usually mainly composed of octanoic acid and decanoic acid. MCTs can be quickly absorbed and transported directly to the liver through the hepatic portal vein, where they are rapidly metabolized and partially converted into ketones, thereby increasing blood ketone levels [85]. Therefore, MCTs are considered ketogenic fats because they increase blood ketone concentrations regardless of calorie or carbohydrate content. The safety of this ketogenic method has also been confirmed [86]. Patients with mild cognitive impairment who take ketogenic beverages containing medium chain triglycerides experience an increase in blood ketone concentration accompanied by improvement in cognitive impairment [87]. Patients with subacute brain injury who received a ketogenic diet and MCT did not experience severe adverse reactions or complications [88]. There is also an experimental method for adding MCT oil to a ketogenic diet to accelerate the induction of ketosis [49, 89]. Stroke mice fed this method performed better in exercise than those on a normal diet [49]. Elevated plasma triglyceride levels are one of the risk factors for stroke, and a formula rich in medium chain triglycerides has been proven to quickly and safely reduce plasma triglyceride levels [90]. Therefore, we can look forward to more studies on MCT improving the prognosis of stroke patients.

## Summary of relevant mechanisms

**Fig. 2:**
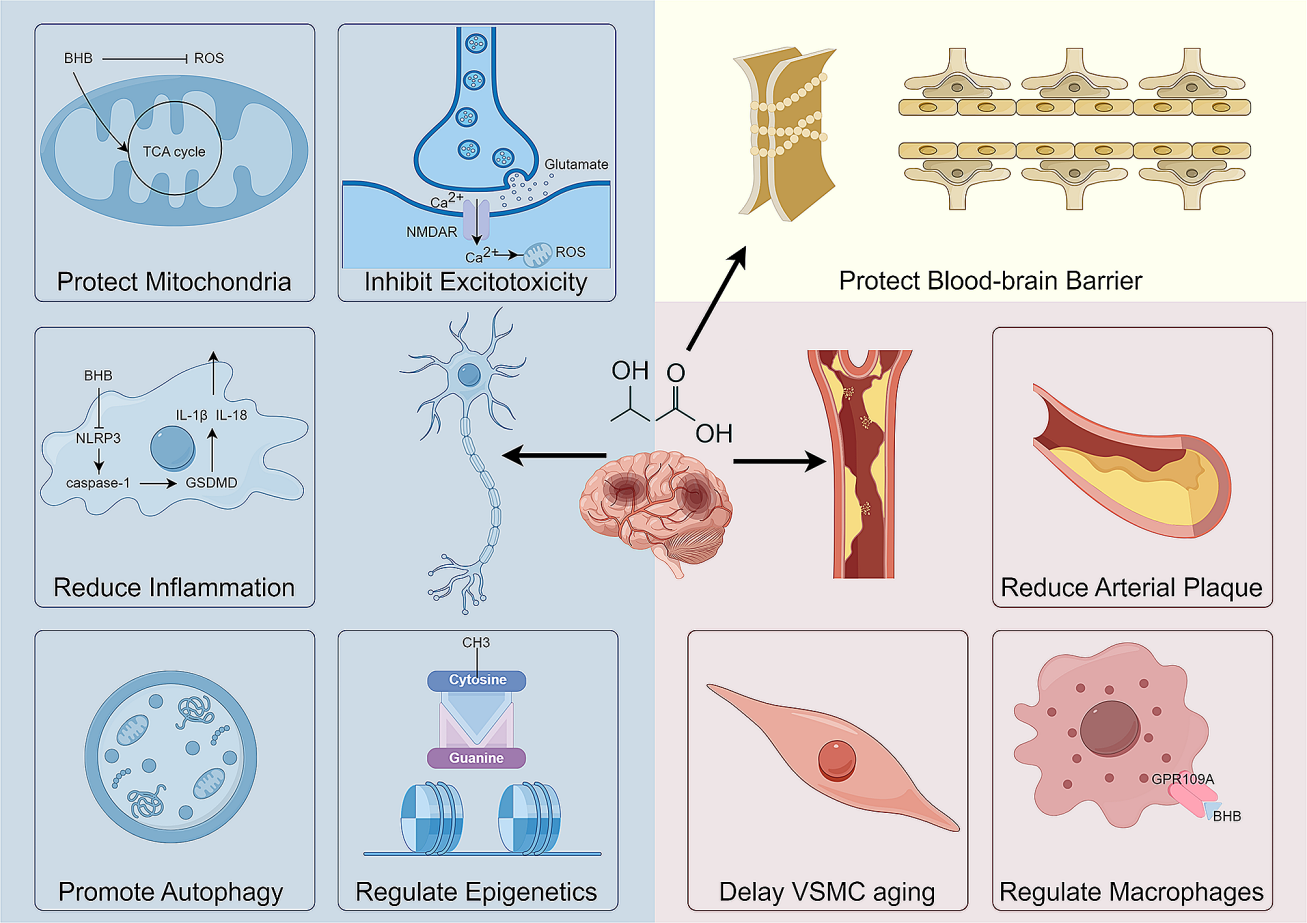
Multidimensional Impact of BHB in Stroke Prevention and Neuroprotection. BHB shields neurons by reducing oxidative stress within mitochondria, preventing excitotoxicity, enhancing autophagy, regulating gene expression through epigenetic mechanisms, and mitigating inflammation. BHB also plays a role in delaying the aging of vascular cells and fortifying the blood-brain barrier, which collectively contributes to its neuroprotective effects in stroke prevention and recovery. This figure was drawn by Figdraw

Thrombotic stroke is the most prevalent type of stroke [91]. The damage caused by focal cerebral ischemia can be divided into irreversible core regions and reversible peripheral tissues, known as ischemic penumbra. Rescuing the neurons in the ischemic penumbra is the focus of our research. Neurons are subjected to a range of pathological processes, including inflammation, excitotoxicity, oxidative stress. These events are intertwined and together drive neurons towards death [92, 93]. BHB modulates these pathological processes, thereby exerting a protective effect on neurons, particularly within the ischemic penumbra. Additionally, its role extends to preserving blood-brain barrier permeability and improving arterial atherosclerosis, contributing to stroke prevention(Fig. 2).

### Impact on neurons

#### Excitotoxicity

Glucose and oxygen deficiency during cerebral ischemia induces glutamate release. Then stimulates Na^+^/Ca^2+^ channels coupled with N-methyl-D-aspartate receptors (NMDARs). The increase in calcium ion influx leads to calcium overload, leading to cell apoptosis, known as excitatory toxicity [94]. BHB continuous infusion reduces NMDA induced brain injury [95]. In a in vivo excitotoxicity model, it has been documented that administration of BHB effectively mitigates neuronal injury and diminishes levels of lipoperoxidation within the rat striatum [96]. In neurons, Stimulation of synaptic NMDARs activates the pro-survival PI3K/Akt signaling pathway, thereby exerting a neuroprotective effect. BHB activated PI3K/AKT/mTOR signaling [97]. Therefore, this signaling pathway may be a key pathway for the neuroprotective effect of BHB on ischemic stroke, but there is no direct research yet. BHB reversed cytotoxicity and the decrease in phosphorylation of ERK and GSK3β induced by the glucose deficiency [98]. The same conclusion was reached for neuronal cells cultured under oxygen and glucose deprivation conditions used to simulate stroke environments [99]. The above effects played a role in reducing cell apoptosis in ischemic stroke mice [100].

#### Oxidative stress and mitochondrial function

After a lack of glucose and oxygen, neuronal oxidative phosphorylation is inhibited, ATP supply is insufficient, mitochondrial membrane depolarization occurs, further triggering release of excitatory amino acids into the extracellular space, intracellular calcium overload occurs, mitochondrial dysfunction occurs, and excessive reactive oxygen species (ROS) is produced. Intraperitoneal injection of BHB into infarct model mice can inhibit ROS driven by glucose metabolism in the infarcted area, ultimately promoting functional recovery [101]. Direct injection into BHB lateral ventricle in MCAO model rats enhanced mitochondrial complex I respiratory chain complex I activity, reduced oxidative stress, inhibited mitochondrial apoptosis, improved neurological scores, and reduced infarct volume after ischemia [99]. In addition to the cerebral infarction model, supplementing BHB to t-MCAO mice can also temporarily improve mitochondrial function [102]. In the study of neurons in vitro, exogenous BHB supplementation can increase the respiratory capacity of neurons [103]. Treating neurons in a low glucose environment with BHB reduced intracellular ROS levels and decreased apoptosis rate [104]. BHB increased mitochondrial membrane potential and increased the ratio, which donated electrons and can be used as a reducing agent to reduce ROS and Sirtuin 3 may have mediated this process. The ways in which Sirtuin 3 reduces cell ROS levels include activating superoxide dismutase 2, activating forkhead box O3a, and catalase [105].

In BHB-induced hippocampal murine neurons, the ratio of single phospholipid to cholesterol was noticeably higher, indicating that the composition of plasma membrane and organelle membrane might change [106]. The pathological and physiological significance of this change still needs further research.

In addition to its role as a metabolic substrate, BHB also has the ability to independently scavenge ROS, possibly due to the presence of hydroxyl groups within it [107]. But recent studies have found that the mechanisms by which the two chiral isomers of BHB reduce ROS levels are different. L-BHB mainly exerts its ability to clear ROS. The metabolism of D-BHB may predominantly protect mitochondrial metabolism, thereby inhibiting the generation of ROS [107, 108].

#### Inflammatory responses and pyroptosis

**Fig. 3:**
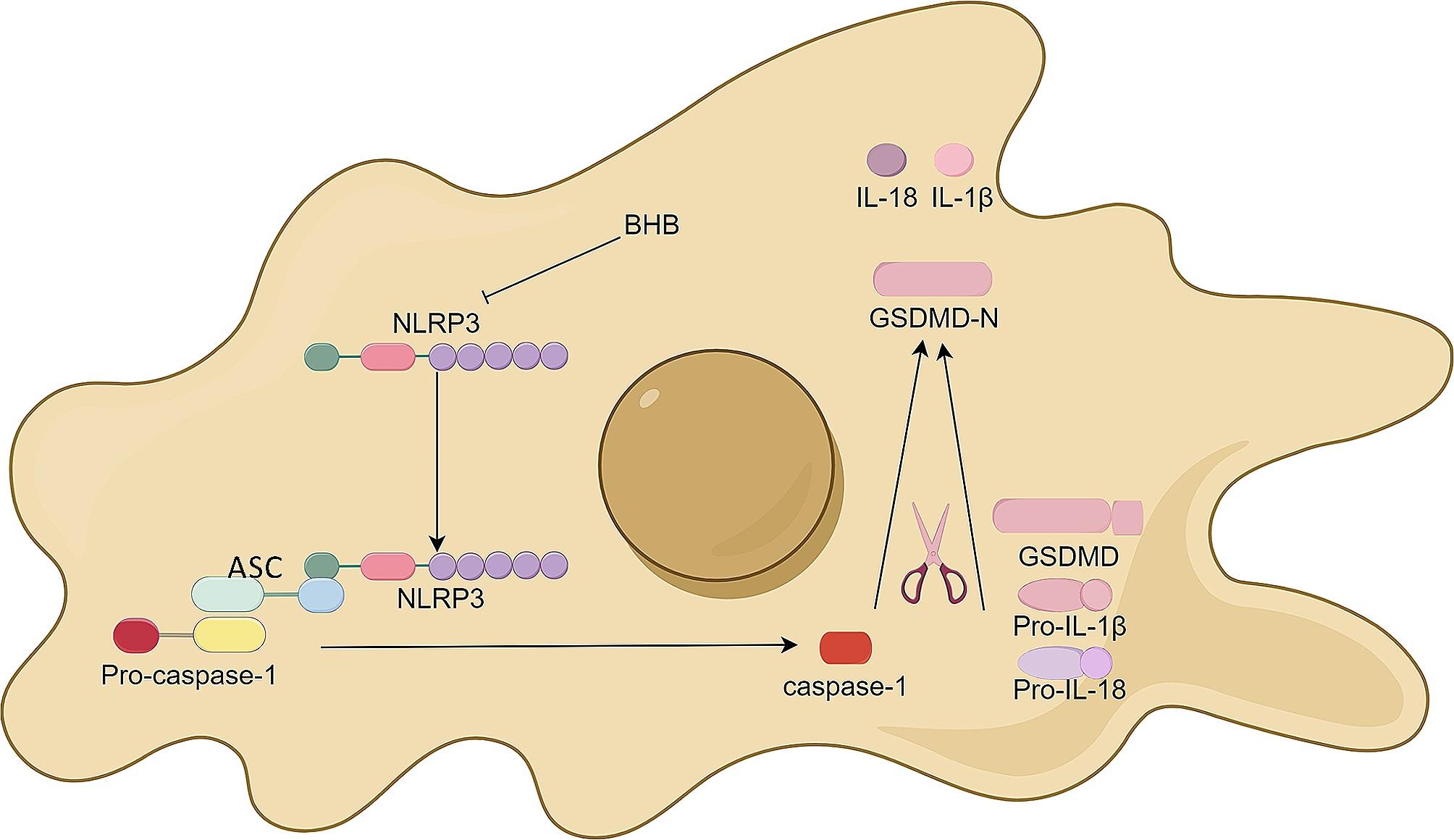
The process of action of BHB and NLRP3 inflammasomes. BHB inhibits the NLRP3 inflammasome activation, potentially through the inhibition of ACS, which seems to be an upstream activator of NLRP3. This figure was drawn by Figdraw

The NLRP3 inflammasome is a major mediator of inflammatory responses during ischemic stroke. The NLRP3 inflammasome is a cytosolic multiprotein complex composed of, inflammatory protease caspase-1, and the innate immune receptor protein NLRP3, a classic nucleotide-binding oligomerization domain-like receptor (NLR) [109]. After stroke, under the environmental conditions of excitatory toxicity and oxidative stress, NLRP3 inflammasomes are activated, promoting the cleavage and maturation of Caspase-1 which cleaves and splits gasdermin D (GSDMD) and pro-interleukin-1β, pro-interleukin-18, forming GSDMD-N and IL-1β,IL-18, suggesting microglia activation [110], mediating nerve cell dysfunction and brain edema and ultimately leading to nerve cell death [111, 112]. Pyroptosis is a novel programmed cell death pathway that relies on caspase-1/4/5/11, characterized by a strong inflammatory response and involvement in the occurrence and development of stroke, first proposed by Cook et al. in 2001 [113]. Cells that undergo pyroptosis show features such as swelling until the cell membrane rupture and release of proinflammatory intracellular contents, DNA damage and cell lysis and eventually cell death. BHB exerts an inhibitory influence on NLRP3 inflammasome activation through multifaceted pathways, as depicted in the following illustration, thereby offering a potential therapeutic strategy for mitigating neuronal injury induced by ischemic stroke (Fig. 3). Based on existing researches, the pathways by which BHB inhibits NLRP3 activation may include potassium channel, hypoxia inducible factor-1α (HIF-1α) and endothelium reticulum (ER) stress.

In monocytes, BHB inhibits the NLRP3 inflammasome by preventing K^+^ efflux and reducing oligomerization and speck formation [114], thereby inhibiting the generation of IL-1β and IL-18 in human mononuclear cells which plays a role in inhibiting inflammation. However, in microglia, researchers did not observe that BHB inhibits the activation of the inflammasome and inhibits IL-1β induced by synuclein fibrils [115]. The body is not only a complex environment comprising various cells but is also influenced by hormones such as insulin. Therefore, the mechanism by which BHB affects the secretion of IL-1β in the human body remains a subject of debate.

Succinate is an intermediate product of BHB catabolism in mitochondria. When it accumulates and enters the cytoplasm, it inhibits proline hydroxylase, leading to the elevation of HIF-1α. Accumulated HIF-1α and IL-10 may mediate the attenuation of NLRP3 inflammasomes, thereby downregulating the pro-inflammatory factor TNF-α and IL-6 expression [116]. A ketogenic diet can achieve the above effect [50, 116]. Intraventricular injection of BHB can also increase the HIF-α level of stroke mice compared to the control group [50]. After using Liquiciguat to suppress HIF-1α expression, the expression level of NLRP3 and IL-1β was significantly reduced, and the neuroinflammatory response and cell apoptosis were also improved [117].

Protein folding is a primary function of the endoplasmic reticulum. After the occurrence of ischemic stroke, the ischemic and hypoxic environment can lead to a decrease in the aforementioned abilities, causing errors in protein folding, leading to ER stress [118]. During this process, GRP78 helps to prevent protein misfolding. The expression of GRP78 in mice on a ketogenic diet after stroke was higher than that in the normal diet group, indicating that endoplasmic reticulum stress was inhibited by elevated ketone bodies [51]. Extracorporeal BHB treatment of oxygen–glucose deprivation/reoxygenation SH-SY-5Y cells, similar to the application of endoplasmic reticulum stress inhibitor tauoursodeoxycholic acid, also played a similar role in inhibiting NLRP3 inflammasomes [51]. Mechanistically, BHB promotes extracellular calcium influx through GPR109A, leading to an increase in intracellular Ca^2+^ levels. The release of Ca^2+^ from the endoplasmic reticulum to mitochondria is reduced, thereby inhibiting ER stress caused by depletion of ER Ca^2+^ storage [119]. Meanwhile, the hydroxycarboxylic acid receptor 2 (HCAR2, GPR109A) may also mediate the neuroprotective effect of BHB in ischemic stroke [120]. Indeed, while both ketogenic diets and BHB significantly decrease infarct size after MCAO, this protective effect is lost in the HCAR2 knockout mice despite higher plasma levels of ketone bodies. The protective effect of HCAR2 activation depended on COX1 and HPGDS, the key enzymes that synthesize PGD2 [120].

The use of BHB in stroke patients is not only beneficial for reducing neuroinflammatory responses, but also more likely to alleviate post-stroke depression. BHB exerts antidepressant-like effects, possibly by inhibiting microglial NLRP3-induced inflammation in the hippocampus [121].

However, whether these inflammation-attenuating effects of ketones can be replicated in humans at physiological concentrations is controversial. Two large clinical studies seem to have reached opposite conclusions. In a randomized crossover trial, oral ketone monoester(12 g BHB) before meals for 14 days in obese people decreased caspase-1 activation, reduced cellular inflammation and enhanced vascular function [83]. In another randomized double-blind placebo-controlled trial targeting healthy subjects, it was found that LPS-induced activation of caspase-1 and maturation of IL-1β were not significantly affected after 30 min of ketone salts (0.3 g BHB per kg) or ketone monoester (0.482 g BHB per kg) [122]. In view of the above difference, we propose the following assumptions: First, the genes involved in mitochondrial autophagy-NLRP3 pathway may affect weight regulation and metabolic control [123]; Second, there are differences in the determination methods of active caspase-1; Third, the expression of NLRP3 in obese individuals is greatly influenced by insulin resistance [124] and it is unknown whether BHB mediates the NLRP3 inflammatory response through the increase of insulin or other hormones or metabolites [125].

#### Autophagy

The increase in AMP/ATP ratio, caused by cerebral ischemia after IS, activates 5’-Adenosine monophosphate-activated protein kinase (AMPK) due to oxygen and glucose deficiency. Activated AMPK inhibits mTORC1 and initiates autophagy. Autophagy is a natural, conserved cellular degradation process that reduces damaged cell debris and organelle accumulation [126]. A major upstream regulator of starvation-induced autophagy is the energy sensor 5′ adenosine AMPKα. Treatment with dorsomorphin, an AMPKα inhibitor, also blocked the starvation-induced increase in BHB. These data suggest that BHB biosynthesis is dependent on AMPKα-induced autophagy [76]. Besides, in the same cell culturing, BHB was reported to promote autophagic flux and reduce neuronal death [127]. The same team found that BHB stimulates autophagy-lysosome pathway through AMPK activation and TFEB-mediated lysosome generation, and SIRT2 may be a target of action worthy of attention [108]. BHB treatment prevented the cleavage of the lysosomal membrane protein and stimulated the autophagic flux in the ischemic core and the penumbra [128]. Not only does the increase in hunger induced BHB generation depend on autophagy, but BHB itself also plays a promoting role in autophagy. Therefore, we can reasonably assume that autophagy-induced anti-inflammatory and antioxidant responses, leading to vascular protection, may be part of the mechanism behind BHB’s neuroprotective effect in hypoxic environments.

#### Epigenetics

The regulation at the epigenetic level does not involve changes in gene nucleotide sequences, but rather regulates the interaction between the environment and genome, mainly including histone post-translational modifications, DNA methylation, and non-coding RNA. Epigenetics has become a hot research area recently [129].

A portion of the insights into the influence of BHB on methylation is derived from the transcriptional regulation of brain-derived neurotrophic factor (BDNF). BDNF, widely expressed in the central nervous system, gut and other tissues, combines with tropomyosin receptor kinase B to play a neuroprotective role in conditions such as neurotoxicity and cerebral ischemia [130]. The BDNF level in stroke patients significantly decreased compared to the healthy control group, and its level decreased with the severity of the stroke [130].

BHB has the ability to cross the blood-brain barrier and aggregate in the hippocampus. Both a ketogenic diet and BHB administration induce the expression of BDNF in the hippocampus. In vitro experiments, it was observed that BHB significantly increased BDNF expression in both primary hippocampal neurons and the hippocampus neuronal cell line HT22, even under adequate glucose supply. The increased BHB levels inhibit HDACs, particularly HDAC2 and HDAC3, leading to an upregulation of BDNF expression [131]. Moreover, BHB stimulation operates through the cAMP/PKA-triggered phosphorylation of CREB and subsequent up-regulation of histone H3 Lysine 27 acetylation (H3K27ac) binding at BDNF promoters I, II, IV, and VI [132]. H3K27ac is an epigenetic label formed by acetylation of lysine residue 27 on histone H3 [133]. It is highly conceivable that BHB may contribute to increasing BDNF levels, thereby playing a role in epigenetic regulation.

### Impact on the blood-brain barrier

The blood-brain barrier (BBB) is a dynamic and tightly connected structure composed of brain microvascular endothelial cells, pericytes, astrocyte foot processes and the basement membrane. The BBB ensures a stable microenvironment for neurons and contributes to the maintenance of cerebral homeostasis [134]. A few hours after the onset of ischemic stroke, the permeability of the blood-brain barrier increases, reactive oxygen species increase, and white blood cell infiltration leads to secondary inflammation, vascular edema, and hemorrhagic transformation of infarction [135]. Therefore, early improvement of blood-brain barrier permeability can play a role in improving the prognosis of stroke patients.

BHB has demonstrated its ability to protect the integrity of BBB. This is demonstrated by its ability to reduce the ultrastructural damage and permeability of the blood-brain barrier, restore the expression of tight junction-related proteins in the hippocampus, and inhibit the expression of Matrix Metalloproteinase-9 [136]. MCC950 can specifically inhibit NLRP3 inflammasomes. Treating oxygen-glucose deprived brain endothelial cells with the above-mentioned drugs reduced the secretion of MMP9 and reduced cell mortality [137]. Due to the previously mentioned inhibitory effect of BHB on NLRP3, we can speculate that the protective effect of BHB on BBB is also influenced by the inhibition of NLRP3. But recently, researchers have also conducted glucose deprivation experiments by inducing pluripotent stem cell-derived brain microvascular endothelial like cells and found that BHB treatment did not improve the blood-brain barrier function or alter its metabolic characteristics in glucose deficient cells [138]. However, in low-sugar environments, BHB can have a protective effect, which may be related to its promotion of the expression of monocarboxylate transporter-1 [138].

### Impact on atherosclerosis

Direct administration of BHB may be a more reliable method for inhibiting atherosclerosis. Research on the mechanism mainly involves cholesterol metabolism and the regulation of endothelial cell function. ApoE-/- mice, when fed a Western diet, serve as the predominant murine models for studying atherosclerosis, with plaque formation serving as a pivotal indicator of atherosclerotic pathology [139]. “Western diet” refers to a typical dietary structure composed of high-fat and high-sugar foods. Even if the diet is high in fat, the exogenously administered BHB reduces plaque volume in ApoE-/- mice, which inspired us about the feasibility about the long-term treatment of atherosclerosis with direct supplementation of BHB. In ApoE-/- mice subjected to a Western diet, whether through regular exercise, direct intraperitoneal injection of BHB [140] or intragastrically administrated with BHB [119], there is a reduction in plaque volume. Compared with the control group, ApoE-/- mice that were fed with a ketogenic diet generated more plaques [141].

Resistin (RSN) is a recognized risk factor for atherosclerosis and might serve as an independent risk marker for ischemic stroke in individuals with type 2 diabetes [142]. Elevated plasma level of RSN appears to be correlated with an augmented risk of 5-year mortality or disability subsequent to atherothrombotic ischemic stroke. The mechanism behind this phenomenon may be that RSN activates microsomal triglyceride transfer protein and induces insulin resistance in liver cells, thereby increasing hepatocyte VLDL apoB and lipid secretion [143]. The treatment of BHB reversed the trend of plaque volume growth in ApoE-deficient mice fed a high-fat diet and a notable decrease was observed in serum RSN level [144]. However, dieting did not have a similar effect [145]. Therefore, the pathway of action of BHB on RSN needs further exploration.

Elevated BHB was found, both in vivo and in vitro, increasing the expression levels of key cellular cholesterol transport proteins within the plaques. This led to a decrease in intracellular lipid deposition, thereby inhibiting the formation of macrophage foam cells and reducing the level of atherosclerosis [140]. In addition, BHB reduced the proinflammatory M1 macrophage proportion and restored homeostasis of cholesterol metabolism by acting on macrophages through GPR109A [119, 146].

In vascular smooth muscle cells(VSMCs), BHB slows the aging process by activating a Lamin B1 pathway [147]. BHB interacts with nuclear ribonucleoprotein hnRNP A1, leading to upregulation of the transcriptional factor OCT4, which causes increased expression of Lamin B1 [148]. Lamin B1 downregulation was reported as a marker of VSMC senescence [149], and Lamin B1 was elevated in atherosclerosis [150].

In animal experiments, a ketogenic diet was administered for 28 days after surgery for common carotid artery injury, and it was found that the KD attenuates neointimal hyperplasia through suppressing oxidative stress and inflammation to inhibit VSMC proliferation and migration [151]. We can make a reasonable guess that BHB improves endothelial and smooth muscle cell function and reduce atherosclerosis.

One of the most intriguing aspects of BHB is its role as an endogenous and selective inhibitor of HDACs IS. By modulating histone acetylation, BHB exerts intricate downstream effects on the transcriptional landscape, influencing genes like Forkhead box O3a and Metallothionein [148]. These genes are implicated in many biological processes, ranging from tumorigenesis and angiogenesis suppression to vascular calcification and inflammation regulation [18, 148, 152–156]. It is worth emphasizing that HDAC9 influences phenotypic characteristics of vascular smooth muscle cells and is connected to atherosclerotic aortic calcification, and BHB is able to downregulate HDAC9 to suppress vascular calcification [18, 147].

Lysine-hydroxybutyrylation is a novel histone modification method first reported in 2016, wherein BHB is covalently attached to lysine ε-amino groups [157]. The impact of this innovative histone modification approach on the context of stroke requires further investigation. While the precise mechanisms of BHB’s epigenetic regulatory activity remain to be fully elucidated, it is clear that administering BHB or increasing their levels through fasting or exercise presents a promising avenue for improving neuronal activity.

Based on the above, while the regulation of BHB after ischemic stroke involves various biological processes across multiple tissues or cells, including neurons, blood vessels, and the blood-brain barrier, certain common biological processes, pathways, or targets merit more attention. Firstly, under ischemic and hypoxic conditions, BHB can modulate cellular metabolic states and further reduce excitotoxicity [99, 100], inhibit mitochondrial dysfunction and ROS production [101, 102], and enhance autophagy [127, 128], thereby alleviating cellular damage. This process is closely related to the AMPK/mTORC pathway, which regulates cellular energy and metabolic balance. BHB also promotes ionic homeostasis in cells. For instance, BHB can inhibit K^+^ efflux in monocytes to regulate their inflammatory levels [114], and it can promote extracellular Ca^2+^ influx via GPR109A, thereby inhibiting endoplasmic reticulum stress [119, 120]. The improvement of ionic homeostasis, hypoxic conditions, and endoplasmic reticulum stress mediated by BHB collectively leads to the inhibition of the NLRP3 inflammasome [50, 51, 114, 116], thus reducing neuronal damage by suppressing inflammation during ischemic stroke. Additionally, BHB has shown selective inhibition of HDAC in both neurons and vascular smooth muscle cells [131, 147], suggesting a potential impact on epigenetics. Therefore, the roles and mechanisms of BHB in ischemic stroke warrant further attention.

## Conclusions and prospects

In conclusion, while BHB presents a promising avenue for both the treatment and prevention of stroke, a concerted effort to bridge the gap between laboratory findings and clinical application is essential. Addressing the current limitations in our understanding of BHB’s neuroprotective mechanisms and optimizing its clinical usage could significantly impact the management and prevention of stroke, ultimately improving patient outcomes.

Despite significant advances, the precise molecular pathways through which BHB exerts its neuroprotective effects in the context of stroke remain inadequately mapped. The challenge lies in translating the protective mechanisms observed in preclinical studies to human models, where the complexity of human physiology and the heterogeneity of stroke pathology can dilute the clear-cut efficacy seen in more controlled environments. Future research should aim to: 1. Establish a clearer systemic understanding of how BHB interacts with key neuroprotective pathways and how these interactions can be optimized to enhance post-stroke recovery. 2. Develop clinical trials designed to rigorously test the efficacy of BHB supplementation, both as a therapeutic intervention post-stroke and as a preventative measure against the development of stroke, with a focus on dosage, timing, and delivery mechanisms. 3. Investigate the role of BHB in modulating immune responses and inflammation post-stroke, as these areas represent critical but underexplored avenues for potential therapeutic intervention.

## Data Availability

Not applicable.

## Abbreviations

AMPK: Adenosine monophosphate activated protein kinase
ASC: Apoptosis associated speck-like protein
BBB: Blood brain barrier
BDNF: Brain derived neurotrophic factor
BHB: β-hydroxybutyrate
D-BHB: D-β-hydroxybutyric acid
ER: Endothelium reticulum
FFAR3: Free fatty acid receptor 3
GPR109A: G protein-coupled receptor 109 A
GSDMD: Gasdermin D
H3K27ac: Histone H3 Lysine 27 acetylation
HCAR2: Hydroxycarboxylic acid receptor 2
HDACs: Class I histone deacetylases
HIF-1α: Hypoxia inducible factor-1α
KD: Ketogenic diet
KE: Ketone esters
L-BHB: L-β-hydroxybutyric acid
MCAO: Middle cerebral artery occlusion
MCT: Medium chain triglycerides
NMDAR: N-methyl-D-aspartate receptor
NMR: Nuclear magnetic resonance
ROS: Reactive oxygen species
RSN: Resistin
VSMC: Vascular smooth muscle cell
